# CpG-ODN Shapes Alum Adjuvant Activity Signaling *via* MyD88 and IL-10

**DOI:** 10.3389/fimmu.2017.00047

**Published:** 2017-02-03

**Authors:** Luciana Mirotti, Ricardo Wesley Alberca Custódio, Eliane Gomes, Florencia Rammauro, Eliseu Frank de Araujo, Vera Lucia Garcia Calich, Momtchilo Russo

**Affiliations:** ^1^Department of Immunology, Institute of Biomedical Science, University of São Paulo, São Paulo, Brazil

**Keywords:** adjuvants, Alum, TLR agonists, CpG-ODN, T helper cells, OVA model, lung inflammation

## Abstract

Aluminum-containing adjuvants usually referred as Alum are considered as T helper type-2 (Th2) adjuvants, while agonists of toll-like receptors (TLRs) are viewed as adjuvants that favor Th1/Th17 immunity. Alum has been used in numerous vaccine formulations; however, its undesired pro-Th2 adjuvant activity constitutes a caveat for Alum-based vaccines. Combining Alum with TLR-dependent, pro-Th1/Th17 adjuvants might dampen the pro-Th2 activity and improve the effectiveness of vaccine formulations. Here, using the ovalbumin (OVA) model of allergic lung inflammation, we found that sensitization with the synthetic TLR9 agonist, which is composed of oligodeoxynucleotides containing CpG motifs adsorbed to Alum, inhibited the development of OVA-induced lung allergic Th2 responses without shifting toward a Th1 pattern. The conversion of T cell immunity from the polarized allergic Th2 response to a non-polarized form by sensitization with OVA/Alum/CpG was dependent on MyD88 signaling in myeloid cells. Notably, sensitization of IL-10-deficient mice with OVA/Alum/CpG resulted in the development of neutrophilic lung inflammation associated with IFNγ production. However, in IL-10/IL-12-deficient mice, it resulted in neutrophilic inflammation dominated by IL-17 production. We conclude that OVA/Alum/CpG sensitization signaling *via* MyD88 and IL-10 molecules results in non-polarized immunity. Conversely, OVA/Alum/CpG sensitization in presence of MyD88 but absence of IL-10 or IL-10/IL-12 molecules results, respectively, in neutrophilic inflammation associated with IFNγ or IL-17 production. Our work provides novel OVA models of lung inflammation and suggests that Alum/CpG-based formulations might be of potential use in anti-allergic or anti-infectious processes.

## Introduction

Adjuvants (from Latin, adjuvare: to help) can be broadly categorized in two major functional groups based on whether their immune activity is dependent or not on toll-like receptor (TLR) signaling. Aluminum-containing adjuvants, usually referred as Alum, are TLR-independent adjuvants used in numerous vaccine formulations such as the triple vaccine (diphtheria, pertussis, and tetanus), human papillomavirus, and hepatitis vaccines (1, 2). Although Alum has been licensed for human vaccines for almost 100 years, studies on the mechanisms of action and signaling underlying its activity are still in progress (1, 3, 4). Alum has been extensively used in the classical ovalbumin (OVA) asthma model because sensitization with this adjuvant provokes strong antigen-induced T helper type-2 (Th2) responses, characterized by infiltration of effector/inflammatory CD4^+^ T cells and eosinophils into the lung and elevated serum IgE levels (5).

Accumulating evidence indicates that adjuvants act in the early stages of the immune response by activating innate immune responses, which are responsible for driving the polarization of naïve CD4^+^ T cells toward effector inflammatory Th2, Th1, or Th17 cells (6). Various mechanisms and pathways have been advanced to explain how Alum potentiates Th2 activity, among them, IL-4 production by splenic Gr-1^+^ myeloid cell type, conversion of soluble antigen to a particulate form, production of IL-1β *via* activation of NLRP3-inflammassome, release of DNA from dying host cells, induction of uric acid, secretion of prostaglandin PGE2, and others [reviewed in Ref. (2, 3)]. By contrast, the molecular mechanisms of TLR signaling are well characterized and involve the activation of two main downstream signaling pathways: the MyD88- and TRIF-dependent pathways. Some TLR agonists such as monophosphoryl lipid A (MPLA), a TRIF-biased TLR4 agonist, or TLR9 agonists, which are composed of oligodeoxynucleotides (ODN) containing CpG motifs (CpG), have been approved for use in humans (4).

A caveat of Alum-based vaccines might be the undesired pro-Th2 adjuvant activity of Alum. It is postulated that targeting the innate immune system by combining multiple stimuli, such as those generated by TLR-dependent and independent adjuvants, might dampen the pro-Th2 activities of Alum and boost the effectiveness of vaccines (2). Indeed, using the OVA asthma model, we have previously shown that sensitization with TLR4 agonists, adsorbed to Alum, dampens the development of OVA-induced allergic lung Th2 responses without inducing a shift toward a Th1 lung inflammation (7). Importantly, we recently showed that adsorption of a TLR4 agonist to Alum-based tetanus toxoid vaccine also dampens toxoid-induced Th2 cellular responses and IgE production, and enhances humoral IgG antibody responses (8). Thus, it appears that antigen sensitization with two types of adjuvants results in a non-polarized cellular immunity that activates and modifies quantitatively and qualitatively B cell antibody help.

Since CpG-ODN, a synthetic TLR9 agonist, is another adjuvant approved for use in humans (4), we extended our work and studied the effect of sensitization to OVA with CpG-ODN, type C, adsorbed to Alum. Cellular immunity was determined by the phenotype of lung-infiltrating effector/inflammatory CD4^+^ T cells after intranasal (i.n.) OVA challenge and humoral immunity was determined in serum by total IgE production and OVA-specific antibody isotypes.

We found that addition of CpG to Alum attenuated dose-dependently the Th2 sensitization in wild-type (WT) mice. The expression of the MyD88 adaptor molecule on myeloid cells was sufficient to mediate the inhibitory effect on Th2 sensitization. Notably, in IL-10-deficient animals, OVA sensitization with Alum/CpG-adjuvant resulted in the development of neutrophilic airway inflammation associated with IFNγ production, while in IL-10/IL-12p40 double-knockout (KO) mice it was associated with IL-17 production.

Our results indicate that OVA sensitization with Alum/CpG formulation induces T helper phenotypes ranging from non-polarized to IFNγ or IL-17-dominated lung inflammation, depending on MyD88 signaling and the cytokine milieu.

Our study highlights the major signaling pathways involved in adjuvant activities of an Alum-based CpG formulation and suggests its potential use for the development of vaccines or immunotherapies aiming at multiple T helper cell effector functions.

## Materials and Methods

### Mice

The 6- to 12-week-old female C57BL/6 or 129 Sv mice were used. C57BL/6 WT, MyD88-KO, IL-10-KO, IL-12p40-KO, IFNγ-KO, and RAG-KO mice on C57BL/6 background were originally purchased from Jackson Laboratories (Bar Harbor, ME, USA). Double KOs IL-10/IL-12p40-KO or IL-12p40/IFNγ-KO mice were generated in our breeding unit. The 129 Sv WT, IFNα/β receptor (IFNα/βR)-KO, and IFNγ receptor (IFNγR)-KO mice on 129 Sv background (9) were provided by Dr. Luiz Fernando Reis (Ludwig Institute for Cancer Research, São Paulo, Brazil).

Mice were kept at specific pathogen-free breeding unit, Institute of Biomedical Sciences (ICB IV-USP), in five animals per cage in a ventilated caging system with filter tops under a laminar flow hood. Twelve-hour light/dark cycle, temperature-controlled rooms, food and water *ad libitum*, cardboard tubes, and towel paper were used for environment enrichment. Mice were treated according to animal welfare guidelines of ICB (Ethic Protocol 081/09) under National Legislation-11.794 Law.

### Reconstitution RAG-KO Mice with Lymphoid Cells

RAG-KO mice received 20 × 10^6^ spleen cells diluted in sterile phosphate buffer saline (PBS) *via* intraperitoneal (i.p.) route from spleen macerates of WT or MyD88-KO mice. Reconstituted RAG-KO mice were used 14 days later.

### Sensitization and Challenge

Mice were subcutaneously (s.c.) sensitized on days 0 and 7 with 4 µg of OVA adsorbed to 1.6 mg of Al(OH)_3_ (Alum) gel in 0.2 mL of PBS, containing or not TLR agonists. TLR4 agonist (LPS from *Escherichia coli* 055:B5) was purchased from Sigma-Aldrich (St. Louis, MO, USA) and TLR9 agonist (CpG-ODN 2395 Class C) from Invivogen (San Diego, CA, USA). The standard dose of TLR ligands was 10 µg. On days 14 and 21, mice were intra-nasally challenged with OVA (10 µg) in 40 µL of PBS. Control mice consisted of naïve non-manipulated animals. Sensitization and challenge were done under anesthesia with ketamine (50 mg/kg) and xylazine (20 mg/kg). Animals were euthanized by inhaled halothane 24 h after the last challenge; samples were collected, unaware numbered, and decoded after analyses.

Endotoxin (LPS) removal from OVA and Alum preparation was performed as previously described (7). Briefly, chicken OVA (Sigma-Aldrich, St. Louis, MO, USA) was diluted in PBS (2 mg/mL) and depleted of the endotoxin activity (measured by Limulus amoebocyte lysate QCL-1000 kit from BioWhittaker, Walkersville, MD, USA), using two to four cycles of Triton X-114 extractions, as described by Aida and Pabst (10). The endotoxin level of purified OVA (2 mg/mL) was below the limit of detection (less than 0.1 EU).

### Serum Samples and Bronchoalveolar Lavage (BAL)

Blood samples were collected by cardiac puncture, centrifuged, and serum stored was at −20°C. BAL was acquired after lung washing with 1 mL of cold PBS *via* the trachea. Total and differential cell counts of BAL fluids were determined by haemocytometer and cytospin preparation stained with Instant-Prov (Newprov, Brazil) or with Panótico-Rápido (Laborclin, Brazil), both stains based on Romanowsky formulation.

### Enzyme-Linked Immunosorbent Assay (ELISA) for Antibodies and Cytokine Determinations

Total mouse IgE was determined by sandwich-ELISA using kit BD OptEIA ELISA Set (BD, San Diego, CA, USA). OVA-specific IgE was determined by adding serum at multiple dilutions to plates with anti-IgE (SouthernBiotech, Birmingham AL, USA). After washing, biotin-labeled OVA was added and revealed with avidin-HRP plus substrate. Internal sample arbitrarily assigned as 1,000 U was used as standard. OVA-specific IgG2a/c were measured by coating the plates with 20 µg/mL of OVA. Serum samples were added at multiple dilutions and revealed with goat anti-mouse IgG2a conjugated to HRP (Invitrogen, San Diego, CA, USA), which also reacts with IgG2c isotype. Purified mouse IgG2a (Southern Biotech) was used as standard. All ELISA were performed in 96-well maxisorp plates (Nunc, NY, USA). Cytokines levels were assayed by sandwich kit ELISA according to the manufacturer’s recommendation (BD Biosciences, PharMingen, San Diego, CA, USA) as previously described (5). Values were expressed as picograms per milliliter deduced from a standards curve of recombinant cytokines ran in parallel. The limits of detection ranged from 5 to 30 pg/mL.

### Flow Cytometry Analysis

Three lobules (superior, middle, and post caval) of right lungs were digested with collagenase IV (2 mg/mL) and DNAse I (1 mg/mL) (Sigma-Aldrich), at 37°C for 30 min. Cell suspension was obtained after erythrocyte lysis. Extra-cellular staining to identify neutrophils and eosinophils was done using fluorochrome-conjugated antibodies (BD, San Diego CA, USA): anti-CD45 (C363.16A), -Gr-1 (RB6-8C5), -siglecF (E50-2440), and anti-major histocompatibility complex class II (MHC-II) (107630) (Biolegend, San Diego, CA, USA). For each sample, at least 1,000,000 events were collected. Cytokine production by lung cells was determined after stimulation of cells *in vitro* with 10 ng/mL PMA (phorbol 12-myristate 13-acetate) and 750 ng/mL ionomicin (Sigma-Aldrich, St. Louis, MO, USA) incubated with GolgiStop (BD-Biosciences, San Diego, CA, USA) for 12 h at 37°C in RPMI-1640 medium. Cell suspensions were stained with fluorochrome-conjugated antibodies against surface markers CD45 (C363.16A), CD3 (145-2C11), and CD4 (RM4-5). Cells were permeabilized and subsequently fixed with Cytofix/Cytoperm kit with GolgiPlug (BD-Biosciences, San Diego, CA, USA). For intracellular cytokine staining, we used antibodies against IL-4, IFNγ, and IL-10 (BD, San Diego, CA, USA). Only viable and non-doublet cells were considered. Cell acquisition was performed on a FACs CANTO II instrument using FACSDiva software (BD, San Diego, CA, USA). For each sample, at least 300,000 events were collected. Pools of three to five samples from the same group were used. Data were analyzed using FlowJo software (Tree Star). The number of cytokine-producing CD4^+^ T cells was calculated based on the total number of viable cells (trypan blue exclusion test) recovered from digested lung lobules. FACS analysis was performed with pooled samples from three to five mice.

### Lung Histopathology

Lungs were perfused with 10 mL of cold PBS through the right ventricle, fixed in 10% PBS-formalin for 24 h and then in 70% ethanol until embedding in paraffin. Five-micrometer sections were stained with hematoxylin/eosin or periodic acid-Schiff for analysis of cell infiltrates and mucus production, respectively. Lung inflammation score was performed in coded samples by two researchers scoring peribronchial and perivascular cellular infiltrates as: 0 (not present); 1 (<20% of the airways affected); 2 (20–40%), 3 (40–60%), 4 (60–80%), and 5 (>80% of the airways affected) arbitrary units (AU). Mucus score was calculated as the percentage (%) of mucus positive bronchi throughout the lung sections. In experiments shown in Figure 7, lung inflammation was scored by measuring the area of peribronchial infiltrate per length of bronchial basal membrane.

### 1-Methyltryptophan (1-MT) Treatments and Determination of Indoleamine 2,3-Dioxygenase (IDO) Enzymatic Activity

1-Methyltryptophan was used to inhibit IDO activity. Three different treatments were performed on OVA/Alum/CpG sensitized animals: (a) 30-day slow release 1-MT polymer pellet (150 mg/pellet) (Innovative Research of America) was inserted under the dorsal skin of mice on day 0, as previously described; (b) 1-MT (10 mg) (Sigma-Aldrich) diluted in PBS (500 mL) was daily injected by i.p. route from day 0 to day 14; or (c) 1-MT (35 mg) was absorbed to OVA/Alum/CpG formulation. To estimate IDO enzymatic activity in lung, the concentration of kynurenine, an IDO metabolite, was measured using a modified spectrophotometric assay (11).

### Statistical Analysis

Statistical analyses were performed using GraphpadPrism (V.5; GraphPad Software, USA). One-way ANOVA followed by Tukey post-test was performed, as appropriate. Differences were considered statistically significant when *p* value ≤ 0.05. Data are presented as mean ± SE.

## Results

### TLR4 or TLR9 Agonists Adsorbed to Alum Prevent Th2 Allergic Sensitization

**Figure 1:**
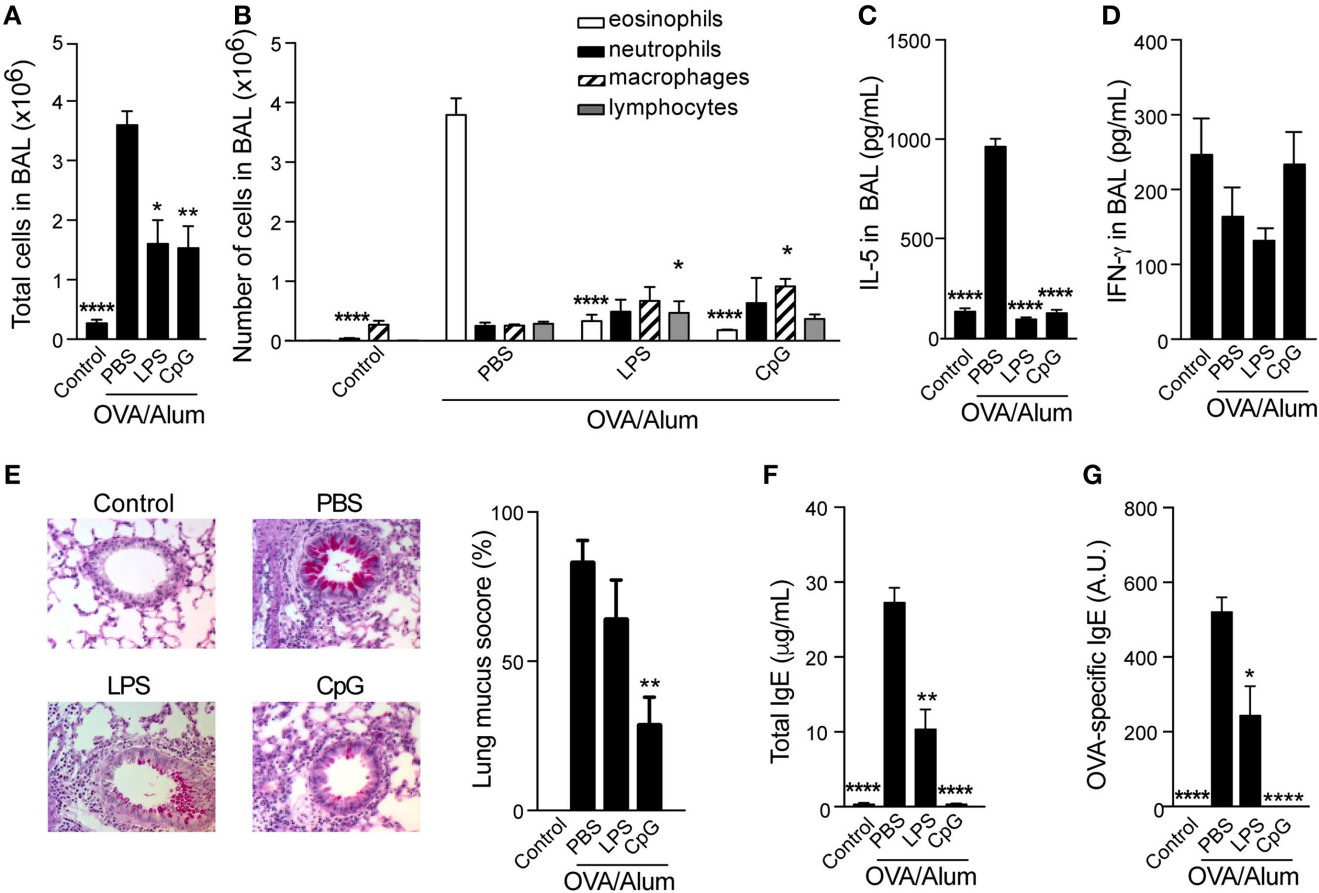
**Toll-like receptors 4 and 9 agonists adsorbed to Alum prevent T helper type-2 allergic responses**. C57BL/6 wild-type mice were sensitized with ovalbumin (OVA)/Alum or with OVA/Alum/CpG or with OVA/Alum/LPS on days 0 and 7 and challenged with OVA on days 14 and 21. Samples were obtained on day 22. **(A)** Total cell and **(B)** differential cell counts in bronchoalveolar lavage (BAL); **(C)** IL-5 and **(D)** IFNγ concentrations in BAL. **(E)** Representative microphotographs of periodic acid-Schiff staining and lung mucus score (see Materials and Methods); **(F)** total IgE and **(G)** OVA-specific IgE serum levels. Control group (*n* = 4) consisted of non-manipulated animals. OVA/Alum/phosphate buffer saline (PBS) group (*n* = 10), OVA/Alum/LPS group (*n* = 5), and OVA/Alum/PBS group (*n* = 5). Values represent the mean ± SD and are representative of three independent experiments. One-way ANOVA: *p < 0.05; **p < 0.01; ****p < 0.0001, different from OVA/Alum/PBS group.

We have previously shown that sensitization to OVA performed with TLR4 agonist adsorbed to Alum prevented the development of asthma-like responses without shifting the lung inflammation toward a Th1 pattern (7). Here, we extended our study and compared the effect of sensitizations with agonists of TLR4 (LPS) or TLR9 (CpG) adsorbed to Alum on the development of OVA-induced airway allergic disease in WT C57BL/6 mice. We found that absorption of LPS or CpG onto OVA/Alum prevented airway allergic inflammation. Decreased number of total cells and eosinophils were observed in BAL fluid (Figures 1A,B) when comparing CpG to allergic group (OVA/Alum). A slight, but significant increase in the number of lymphocytes or macrophages was observed, respectively, in LPS and CpG groups (Figure 1B). The number of infiltrating neutrophils was low and not significantly affected by TLR agonists. The levels IL-5 were significantly decreased in LPS or CpG groups, but the levels of IFNγ production in BAL were not affected (Figures 1C,D). Particularly, significant inhibition of mucus production was only observed in CpG group when compared to allergic group (Figure 1E). Finally, LPS or CpG suppressed total and OVA-specific IgE production when compared to allergic group, being CpG superior to LPS in suppressing IgE production (Figures 1F,G). We conclude that CpG is more effective than LPS in dampening OVA-induced Th2-mediated allergic responses.

### CpG Adsorbed to Alum Attenuates OVA-Induced Airway Allergic Responses in a Dose-Dependent Manner

**Figure 2:**
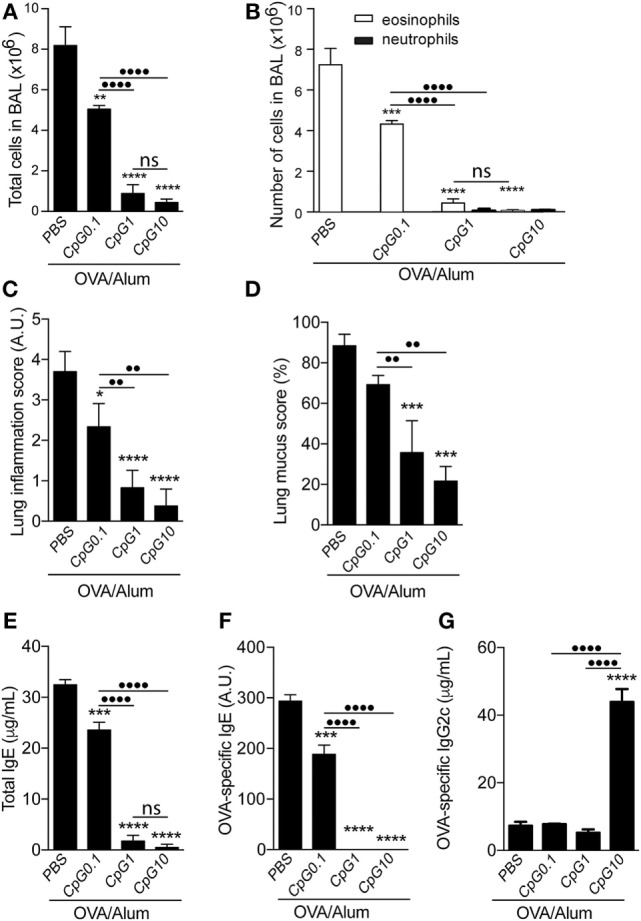
**TLR9 agonist (CpG) attenuates airway allergic responses in a dose-dependent manner**. C57BL/6 wild-type mice were sensitized with ovalbumin (OVA)/Alum or with CpG at different doses (0.1, 1, or 10 μg/animal) absorbed to OVA/Alum on days 0 and 7 and challenged with OVA on days 14 and 21. Samples were obtained on day 22. **(A)** Total cell and **(B)** differential cell counts in bronchoalveolar lavage (BAL); **(C)** lung inflammation score by hematoxylin/eosin staining and **(D)** lung mucus score by periodic acid-Schiff staining (see Materials and Methods); **(E)** total IgE, **(F)** OVA-specific IgE, and **(G)** OVA-specific IgG2c serum levels. The percentage of lymphocytes in BAL was less than 10% in all groups. Values represent the mean ± SD of one experiment. One-way ANOVA: *p < 0.05; **p < 0.01; ***p < 0.001; ****p < 0.0001, different from OVA/Alum/phosphate buffer saline (PBS) group (*n* = 5). One-way ANOVA: ••*p* < 0.01; ••••*p* < 0.0001, different between groups (*n* = 5).

We next evaluated the effect of different doses of CpG on the development of OVA-induced airway allergic disease. WT C57BL/6 mice were sensitized to OVA adsorbed to Alum with or without CpG, at doses of 0.1, 1, or 10 µg. Figure 2 shows that the inhibitory effect of CpG on Th2 responses was dose-dependent and even at low CpG dose (0.1 µg), the number of total cells and eosinophils in the BAL were reduced (Figures 2A,B) as well as lung inflammation and mucus production scores (Figures 2C,D). Regarding IgE antibody production, we found that the higher the dose of CpG the better the inhibition of total and OVA-specific IgE production (Figures 2E,F). OVA-specific IgG2c, a Th1-associated isotype, was significantly increased at high dose of CpG (Figure 2G). Based on the number of total and differential cells (Figures 2A,B) and lung inflammatory score (Figure 2C), we could not detect any deviation toward Th1-type lung inflammation. Overall, adsorption of CpG to Alum was effective in dampening the development of Th2-mediated responses and modifying qualitatively humoral immunity without inducing a noticeable lung inflammation.

### Adsorption of CpG-ODN to OVA/Alum Is Required for Optimal Inhibition of Allergic Sensitization

**Figure 3:**
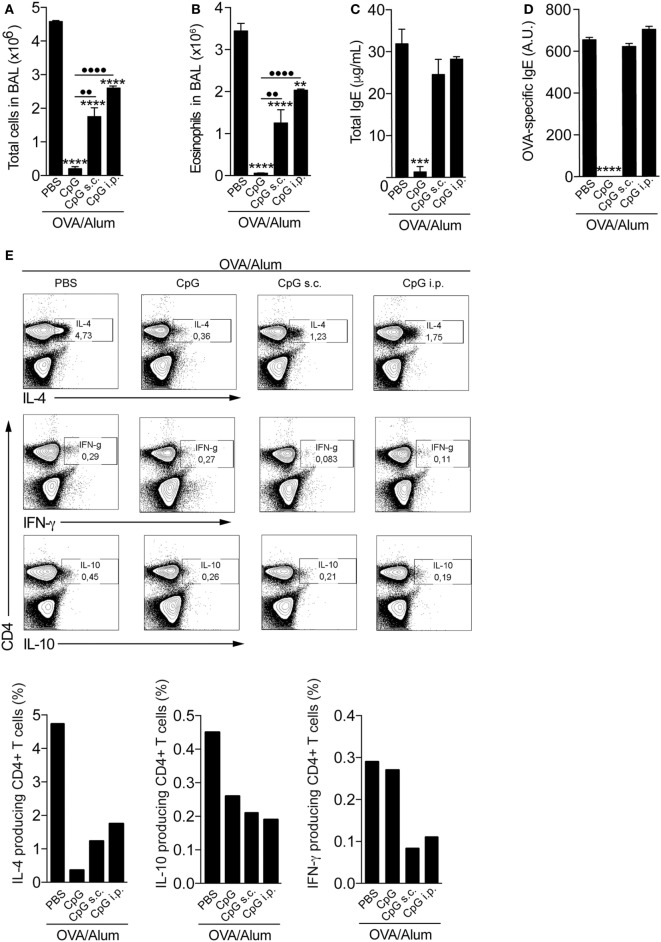
**Optimal inhibition of allergic sensitization by CpG requires adsorption to Alum**. C57BL/6 wild-type mice were sensitized with ovalbumin (OVA)/Alum [phosphate buffer saline (PBS)] or with OVA/Alum plus CpG absorbed to OVA/Alum (CpG) or given by s.c. (CpG s.c.) or by intraperitoneal (i.p.) route (CpG i.p.) on days 0 and 7 and challenged with OVA on days 14 and 21. Samples were obtained on day 22. **(A)** Total cell and **(B)** eosinophil cell counts in bronchoalveolar lavage (BAL); **(C)** total IgE and **(D)** OVA-specific IgE serum levels; **(E)** FACS analysis of cytokine-producing CD4^+^ T cells performed with pooled lung cells from three animals. Values represent the mean ± SD of one experiment. One-way ANOVA: *p < 0.05; **p < 0.01; ***p < 0.001; ****p < 0.0001, different from OVA/Alum/PBS group (*n* = 5). One-way ANOVA: ••*p* < 0.01; ••••*p* < 0.0001, different between groups (*n* = 5).

To evaluate whether the CpG effect on allergic sensitization depends on adsorption to Alum, we compared the effect of CpG in animals sensitized with CpG adsorbed to OVA/Alum to animals that received separately CpG by subcutaneous (on the opposite site of OVA/Alum injection) or i.p. routes. PBS (allergic) group was used as a positive control. We found that optimal inhibition of allergic sensitization by CpG when compared to PBS group requires its absorption to Alum (Figure 3). Specifically, the number of total cells and eosinophils in BAL of mice sensitized with CpG adsorbed to Alum (CpG group) was more reduced than in animals that received CpG separately by s.c. or i.p. routes (CpG s.c. and CpG i.p. groups) when compared to PBS group (Figures 3A,B). Notably, IgE production and OVA-specific IgE antibodies were only significantly reduced in animals sensitized with CpG absorbed to OVA/Alum (Figures 3C,D). FACS analysis of infiltrating lung CD4^+^ T cells producing-cytokines showed that CpG administration dampened Th2 responses in all CpG groups, as revealed by the decreased percentage of IL-4-producing T cells when compared to PBS group (Figure 3E) and confirmed the superior efficacy of CpG absorbed to Alum in inhibiting Th2 sensitization since the CpG group showed the lowest value IL-4-producing T cells when compared to other groups (Figure 3E). Importantly and in line with previous results, the percentage of IFNγ-producing T cells in all CpG groups did not increase when compared to PBS group (Figure 3E) while the percentage of IL-10-producing T cells was lower in all CpG groups when compared to allergic (PBS) group (Figure 3E). Overall, these results clearly indicate that CpG adsorbed to Alum is more effective in dampening the development of allergic responses than when given separately (in a non-absorbed form) during OVA/Alum sensitization. In addition, the results obtained with lung-infiltrating CD4^+^ cytokine-producing T cells (IL-4 versus IFNγ or IL-10) reinforce the view that OVA/Alum/CpG sensitization inhibits Th2 responses without inducing immune deviation toward Th1 pattern or regulatory mechanisms.

### Attenuation of Allergic Sensitization by CpG Requires Signaling through MyD88 Pathway but Not IL-12/IFNγ Axis

**Figure 4:**
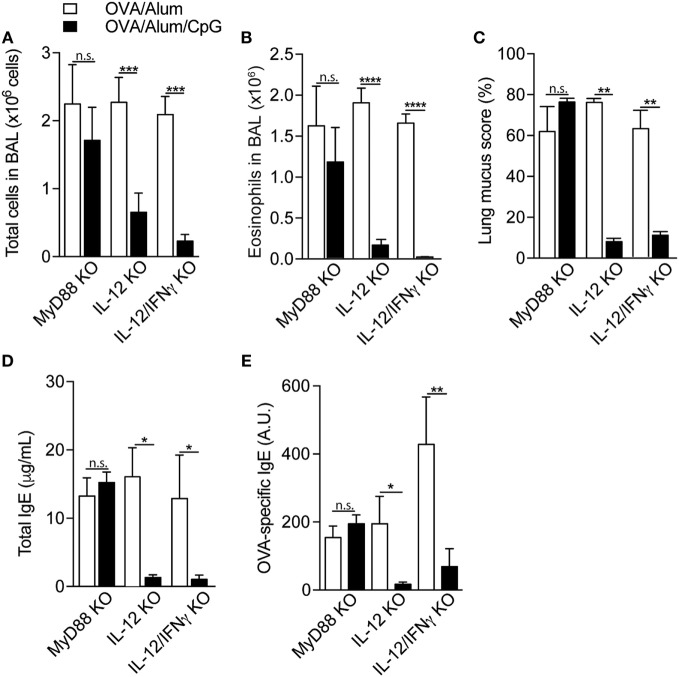
**Attenuation of allergic sensitization by CpG requires signaling through MyD88 pathway but not IL-12/IFNγ axis**. MyD88-knockout (KO), IL-12-KO, or IL-12/IFNγ-KO mice were sensitized with ovalbumin (OVA)/Alum or with OVA/Alum/CpG on days 0 and 7 and challenged with OVA on days 14 and 21. Samples were obtained on day 22. **(A)** Total cell and **(B)** eosinophil cell counts in bronchoalveolar lavage (BAL); **(C)** lung mucus score by periodic acid-Schiff staining; **(D)** total IgE and **(E)** OVA-specific IgE serum levels. Values represent the mean ± SD and are representative of two experiments. One-way ANOVA: n.s. (non-significant), *p < 0.05; **p < 0.01; ***p < 0.001; ****p < 0.0001, difference between OVA/Alum and OVA/Alum/CpG (*n* = 5).

It is well established that CpG signals through MyD88 pathway (12), although it was recently shown that TRIF pathway could also be involved (13). *In vivo* and *in vitro* models have shown that CpG induce the release of type 1 cytokines, notably IL-12 and IFNγ (14), that in turn, downregulate type-2 immune responses (15, 16). To determine whether MyD88 pathway and type 1 cytokines were involved in the CpG-induced inhibition of allergic sensitization, we studied the effect of CpG in MyD88-KO or IL-12p40-KO or IL-12p40/IFNγ double-KO mice. We first found that OVA/Alum sensitization induced significant airway allergic inflammation in MyD88-KO, IL-12-KO, or IL-12/IFNγ double KOs mice, as shown by increased number of total cells, eosinophils in the BAL, lung mucus production, and total and OVA-specific IgE antibody production (Figures 4A–E). Control mice did not show these alterations (data not shown). Absorption of CpG to Alum (OVA/Alum/CpG group) inhibited the development of allergic responses in all KO mice, except MyD88-KO mice (Figures 4A–E). These findings indicate that the expression of MyD88, but not IL-12/IFNγ molecules, is essential for the inhibition of allergic sensitization by CpG. Besides IFNγ, type I interferons (IFNs) have also been proposed as mediators of the immune modulatory effects of CpG (17). Studies on murine models of allergic airway inflammation have shown that IFN-α and IFN-β downregulate eosinophil and CD4^+^ T cell recruitment into airways (18, 19). Therefore, to extend our results we also included in our study IFNα/βR-KO and IFNγR-KO mice. We found that addition of CpG to OVA/Alum reduced airway eosinophilic inflammation, IL-5, and IgE production (Figures S1A–E in Supplementary Material), indicating that IFNαβ or IFNγ are not critical in the CpG-induced attenuation of allergic responses. The induction of IDO enzyme activity is another pathway that has been proposed for the inhibition of experimental asthma by CpG (13, 20). Therefore, we treated OVA/Alum/CpG sensitized animals with 1-MT, an inhibitor of IDO activity, and also monitored IDO activity by measuring kynurenine levels in lung tissue. As shown in Figure S2 in Supplementary Material, the inhibitory effect of CpG on Th2 immunity was preserved in animals treated with 1-MT when compared to untreated animals. In addition, no augmented IDO activity was detected in the lung of OVA/Alum/CpG group (Figure S2 in Supplementary Material). Altogether, these results indicate that the role of CpG in inhibiting allergic process does not involve type I or type II IFN receptors or IDO activity.

### MyD88 Expression on Myeloid Cells Is Sufficient for Inhibition of Allergic Sensitization by CpG

**Figure 5:**
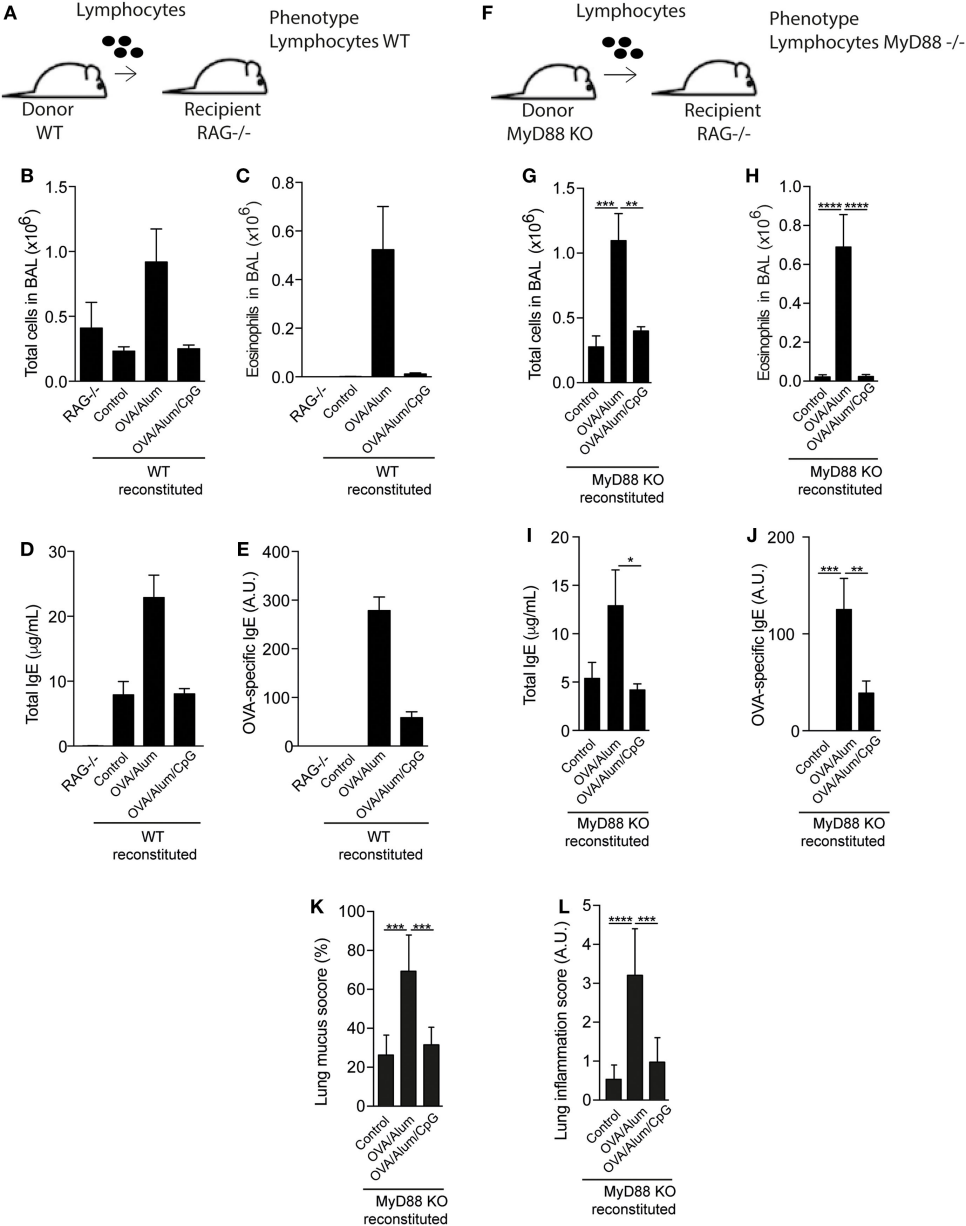
**MyD88 expression on adaptive lymphoid cells is not required for inhibition of allergic sensitization by CpG**. **(A)** RAG^−/−^ mice on C57BL/6 background were reconstituted or not with 20 × 10^6^ spleen cells of C57BL/6 wild-type (WT) or MyD88-knockout (KO) mice. Fourteen days later, mice were sensitized with ovalbumin (OVA)/Alum or OVA/Alum/CpG on days 0 and 7 and challenged with OVA on days 14 and 21. Samples were collected on day 22. **(A,F)** Schematic protocol of reconstituted RAG^−/−^ mice with WT or MyD88-KO spleen cells; **(B,G)** total cell and **(C,H)** eosinophil cell counts in bronchoalveolar lavage (BAL); **(D,I)** total IgE and **(E,J)** OVA-specific IgE serum levels; **(K,L)** lung mucus (periodic acid-Schiff) and inflammation (hematoxylin/eosin) scores (see Materials and Methods); RAG^−/−^ group represents non-manipulated RAG^−/−^ mice. Control group represents reconstituted RAG^−/−^ mice without further manipulation. Values represent the mean ± SD and a representative of three independent experiments. One-way ANOVA: *p < 0.05; **p < 0.01; ***p < 0.001; ****p < 0.0001, compared to OVA/Alum group (*n* = 7).

Since MyD88 molecule was critical for the inhibitory effect of CpG, we next determined the role of MyD88 expression on myeloid or adaptive lymphoid cells. For this, we first reconstituted RAG-1-KO mice, which express MyD88 molecule on myeloid cells but lack adaptive mature B and T lymphocytes (21) with spleen cells of WT or MyD88-KO mice as depicted in Figures 5A,F. Reconstituted RAG-KO mice with WT spleen cells and sensitized with OVA/Alum developed allergic eosinophilic inflammation, showing increased total and eosinophils cell counts in BAL (Figures 5B,C) as well as increased levels of total and specific IgE, when compared to non-reconstituted (RAG^−/−^) or reconstituted but non-manipulated (Control) groups (Figures 5D,E). By contrast, mice sensitized with OVA/Alum/CpG did not develop airway allergic inflammation (Figures 5B,C) and showed decreased IgE production when compared to OVA/Alum group (Figures 5D,E). As expected, non-reconstituted (RAG^−/−^) mice have no serum IgE (Figure 5D). RAG-KO mice reconstituted with MyD88-KO lymphoid cells and sensitized with OVA/Alum also developed allergic eosinophilic inflammation (Figures 5G,H) as well as increased levels of total and OVA-specific IgE antibodies when compared to control groups (Figures 5I,J). Importantly, OVA/Alum/CpG group did not develop airway allergic inflammation and IgE production was decreased when compared to OVA/Alum group (Figures 5G–J). Lung mucus and inflammation scores confirmed the inhibitory effect of CpG on lung allergic inflammation in reconstituted RAG-KO mice with MyD88-KO lymphoid cells (Figures 5K,L). Therefore, CpG signaling through MyD88 pathway on myeloid cells was sufficient to inhibit Alum Th2 sensitization.

### OVA/Alum/CpG Sensitization of IL-10-KO or IL-10/IL-12-KO Mice Induces Neutrophilic Airway Inflammation Dominated, Respectively, by IFNγ or IL-17 Cytokines

**Figure 6:**
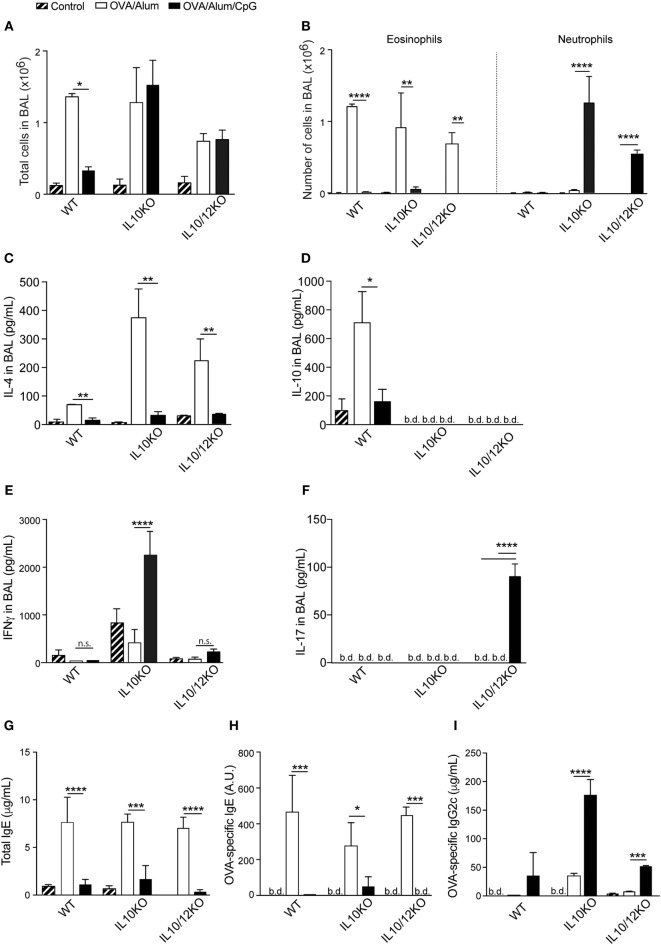
**Alum-based CpG sensitization induces, respectively, in IL-10-knockout (KO) or IL-10/IL-12-KO mice airway inflammation dominated by IFNγ or IL-17**. C57BL/6 wild-type and IL-10-KO mice were sensitized with ovalbumin (OVA)/Alum or with OVA/Alum/CpG on days 0 and 7 and challenged with OVA on days 14 and 21. Samples were obtained on day 22. **(A)** Total cell counts and **(B)** eosinophil and neutrophil numbers in bronchoalveolar lavage (BAL); **(C)** IL-4, **(D)** IL-10, **(E)** IFNγ, and **(F)** IL-17 in BAL; **(G)** total IgE, **(H)** OVA-specific IgE, and **(I)** OVA-specific IgG2c serum levels. Values represent the mean ± SD and are representative of two independent experiments. One-way ANOVA: *p < 0.05; **p < 0.01; ***p < 0.001; ****p < 0.0001, difference between OVA/Alum and OVA/Alum/CpG (*n* = 5).

Our results indicated that type 1 cytokines or respective receptors were not essential for the inhibition of allergic sensitization induced by CpG. Since CpG signaling through MyD88 also triggers the production of IL-10, a molecule that has been shown to suppress both Th1 and Th2 responses (22), we turned our focus to the role of IL-10 in our model. For this, animals were sensitized with OVA/Alum or OVA/Alum/CpG and the results obtained in IL-10 KO mice were compared with those obtained in WT mice. Both mouse strains developed allergic inflammation when sensitized with OVA/Alum as revealed by total and eosinophil cell counts in BAL (Figures 6A,B). However, an apparent difference was found between WT and IL-10-KO mice sensitized with OVA/Alum/CpG, since WT mice did not develop airway inflammation while IL-10-KO mice developed an intense recruitment of inflammatory cells in BAL that was predominantly constituted of neutrophils (Figures 6A,B). All CpG groups showed lower IL-4 production than OVA/Alum groups (Figure 6C) while the levels of IL-10 were increased in allergic (OVA/Alum) group but not in OVA/Alum/CpG groups and as expected, they were bellow detection in IL-10-KO mice (Figure 6D). Importantly, in OVA/Alum/CpG groups, high levels of IFNγ in BAL were only found of IL-10-KO but not in WT mice (Figure 6E). IL-17 levels in BAL were bellow detection in all groups (Figure 6F).

These results indicate that, in absence of IL-10, sensitization with OVA/Alum/CpG instead of inducing non-polarized immunity results in the development of polarized IFNγ-dominated lung inflammation. Because IL-12 is pivotal to induce Th1 immunity, we also determined the inflammatory phenotype of IL-10/IL-12p40 double-KO mice. Double-KO animals sensitized with OVA/Alum also developed allergic airway inflammation as revealed by total and eosinophil cell counts in BAL (Figures 6A,B). Eosinophil cell counts were decreased in CpG group when compared to OVA group (Figure 6B), but CpG group also showed increased number of neutrophils in BAL (Figure 6B). Notably IL-17, but not IFNγ production was significantly increased only in CpG group of IL-10/12-KO mice when compared to OVA/Alum groups (Figures 6E,F). These results indicate that in IL-10 KO mice IFNγ predominate while in IL-10/12-KO mice IL-17 predominate in BAL of CpG groups. As shown before, IgE production was inhibited while IgG2c production was increased in CpG groups when compared to allergic (OVA/Alum) group (Figures 6G,H) and in IL-10-KO mice, the production of OVA-specific IgG2c antibodies reached the highest levels (Figure 6I).

**Figure 7:**
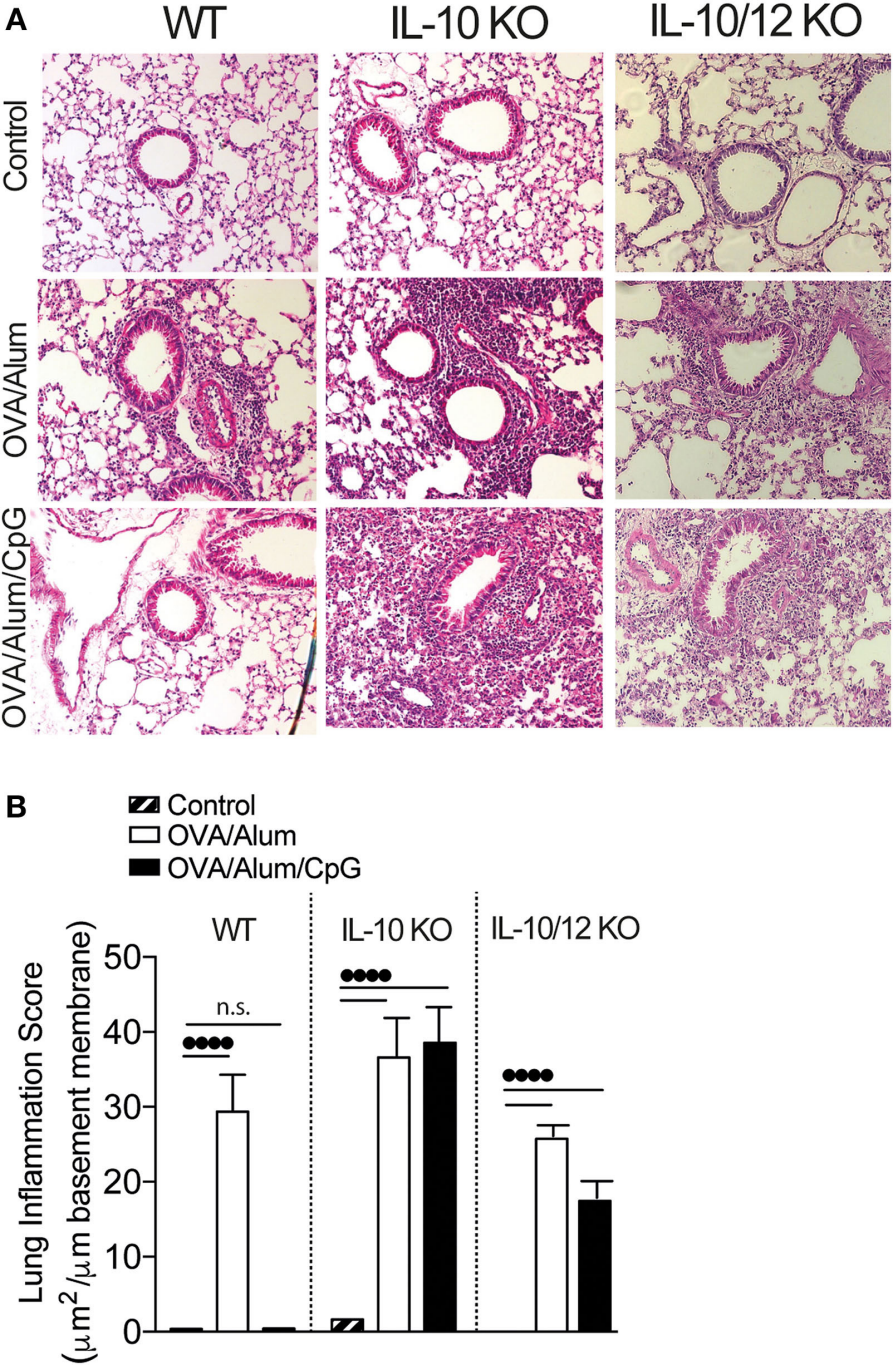
**Lung histology and lung inflammation score of wild-type (WT), IL-10-knockout (KO), or IL-10/IL-12-KO mice**. C57BL/6 WT, IL-10-KO, or IL-10/IL-12 double-KO mice were sensitized with ovalbumin (OVA)/Alum or with OVA/Alum/CpG on days 0 and 7 and challenged with OVA on days 14 and 21. Samples were obtained on day 22. Control group consisted of non-manipulated animals. **(A)** Representative microphotographs of lung sections stained with hematoxylin/eosin and **(B)** lung inflammation score (see Materials and Methods); values represent the mean ± SD and are representative of two independent experiments. One-way ANOVA: ••••*p* < 0.0001, different between groups (*n* = 5).

The above results indicate that phenotype of adaptive immunity to OVA resulting from sensitization with OVA/Alum/CpG varies depending on the presence of IL-10 and IL-12 cytokines. In WT mice, intranasal OVA challenge does not results in lung inflammation while in IL-10 KO or IL-10/IL-12-KO mice intranasal OVA challenge induces airway neutrophilic inflammation and lung inflammation associated with IFNγ or IL-17 production. In order to reveal and quantify lung inflammation, we performed histological analysis of lung sections of Control, OVA/Alum, and OVA/Alum/CpG groups in WT, IL-10-KO, and IL-10/12-KO mice. We found that all OVA/Alum sensitized strains developed peribronchial inflammatory infiltrates when compared to respective Control groups as shown by representative micrographs of lung section and lung inflammation scores (Figures 7A,B). In WT mice, lung inflammation of mice sensitized with OVA/Alum/CpG was absent and similar to Control group (Figures 7A,B) while in IL-10-KO or IL-10/12-KO mice sensitized with OVA/Alum/CpG the intensity of lung inflammation was significantly higher when compared to Control group (Figures 7A,B). Therefore, we conclude that besides MyD88, IL-10 is another molecule that plays a critical role in shaping Alum/CpG-adjuvant activity.

## Discussion

In the present study, using an OVA model of lung inflammation, we investigated the phenotype of adaptive immunity to OVA resulting from sensitizations with an Alum-based formulation combined or not with CpG. We found that OVA/Alum sensitization always resulted OVA-induced eosinophilic lung inflammation, which intensity varied from experiment to experiment, but that upon absorption of CpG to Alum invariable inhibited its pro-Th2 adjuvant activity and the consequent development of allergic lung inflammation, without shifting toward Th1 immunity. These results confirm and extend our previous work with TLR4 agonists (7, 8) and in the light of the hygiene hypothesis, we postulate that this type of immunological stimuli might be operating in individuals exposed to allergens and microbial products. Indeed, it has been shown that school children with higher mattress concentrations of muramic acid, a broad marker of microbial exposure, had significantly lower prevalence of allergy (23).

It has been shown that CpG stimulates dendritic cells (DCs) to produce IL-12, which, in turn, induce effector Th1 cells (15). Likewise, the inhibitory effect of CpG in asthma models has been associated with the stimulation of Th1-type response (24). However, our findings do not support this view since we showed that Th2 inhibition by CpG was preserved in animals deficient in developing Th1 immunity, such as IL-12-KO, IL-12/IFNγ-KO, or IFNγ receptor-KO mice. This is in line with previous work of Kline et al. showing that Th1 cytokines were not required for the suppression of Th2-like immune responses (25). We also investigated whether the inhibition of allergic sensitization could be mediated by type I IFNs, produced upon CpG stimulation and known to exert inhibitory activity on allergic inflammation (13, 18). However, this was not the case since CpG also inhibited allergic inflammation in type I IFN receptor-KO mice.

Activation of the enzyme IDO by CpG was another mechanism proposed for the inhibition of allergic asthma (20). Recently, it was shown that CpG signaling through the TRIF pathway inhibited allergic bronchopulmonary aspergillosis *via* IDO activity (13). However, in our model, the inhibition of Th2 sensitization by CpG was preserved in animals treated with 1-MT, an inhibitor of IDO activity. In addition, IDO activity was not increased in the lungs of CpG-treated animals. We did not investigate the involvement of the TRIF pathway, because we found that MyD88 signaling was essential for CpG-mediated inhibition of allergic sensitization. In addition, experiments with mice lacking adaptive B and T lymphocytes (RAG-KO and MyD88^+/+^) reconstituted with MyD88-KO lymphoid cells revealed that the expression of MyD88 on myeloid cells was sufficient to prevent Th2-mediated responses. Regarding the role of MyD88 and TRIF pathways of TLR signaling in adjuvant activity, Longhi et al. found that poly IC, a TLR3 agonist that signals *via* the TRIF pathway, was a superior adjuvant than CpG in a model of DC-targeted HIV gag protein vaccine in mice for development of Th1 CD4^+^ T cell responses (26). However, our results with CpG and those previously reported by us with MPLA, a TRIF-biased TLR4 agonist or with poly IC adsorbed onto Alum emphasize the role of the MyD88 pathway in preventing Th2 sensitization (7, 8). Thus, it appears that different immunological mechanisms are operating depending on whether the antigen is directly targeted to DCs or adsorbed onto Alum/TLR agonist-based formulations.

Several reports support a major influence of TLR signaling in antigen presentation by DCs and regulation of antigen immunogenicity. In line with our work, it was shown that TLR ligands could activate DCs to release an uncharacterized MyD88-dependent negative signal that impairs Th2 cell development (27). It has been demonstrated *in vitro* that TLR controls the generation of T cell receptor ligands derived from endocytosed cargo, suggesting that TLR signaling *via* MyD88 in DC affects the antigen presentation process (28–30). We anticipate that Alum might function as a physical depot for slow release of a complex particulate matter containing CpG and OVA, allowing a continuous and/or differential modulation of antigen presenting cell activity that prevent the generation of necessary signals to accomplish Th2 sensitization. In the same vein, Yarovinsky et al. showed that selective responsiveness to a protein from *Toxoplasma gondii*, which signals thorough TLR11, required both TLR signaling and MHC-II recognition acting in *cis* for the induction of IFNγ-secreting CD4^+^ T cells (31). It is noteworthy that, in our model, impairment of Th2 sensitization was more evident when CpG was adsorbed onto Alum than when it was administered separately from OVA/Alum as a bolus by s.c. or i.p. routes. Our results are in agreement with the work of Jankovic et al., which showed that absorption of IL-12 to Alum was more effective than IL-12 administered separately to induce Th1 responses against the HIVgp-120 envelope protein (32). Although antigen sensitization with exogenous IL-12 absorbed to Alum drives the immune response toward Th1 immunity, in our model sensitization with OVA/Alum/CpG resulted in a non-polarized (Th0-like) pattern. We reasoned that the concomitant induction of IL-10 by CpG could be curbing the development of polarized effector Th1 cells. Keeping with this hypothesis, it has been shown that the prevention of bleomycin-induced pneumopathy by CpG treatment was critically dependent on IL-10 production (33). Therefore, we evaluated the role of IL-10 in our model in order to explain why sensitization with CpG did not result in Th1 immunity. We found that IL-10 was essential to prevent OVA-induced lung Th1 immunity since sensitization with OVA/Alum/CpG in IL-10-KO mice resulted in an intense airway neutrophilic inflammation, augmented levels of IFNγ in BAL, and increased number of infiltrating lung CD4^+^ T cells producing IFNγ. In addition, we showed that sensitization with OVA/Alum/CpG in absence of IL-10/IL-12 results in the recruitment of Th17 effector/inflammatory T cells into the lung. It is important to distinguish the role of IL-10 in the sensitization phase versus effector phase. We showed that during the effector phase, IL-10 is associated with Th2-mediated airway inflammation while during the sensitization phase IL-10 production inhibits the development of effector T cells and as consequence OVA challenge does not result in airway inflammation. However, in IL-10-KO mice, OVA/Alum/CpG sensitization induces effector T cells that after OVA challenge are recruited to the lung and mediate airway inflammation dominated by IFNγ production (Th1-like). Therefore, it appears that sensitization with OVA/Alum/CpG does not primes for Th2 immunity but for Th1/Th17-like immunity that is not developed due to the regulatory/inhibitory effect of IL-10. Notably, sensitization with CpG in WT mice did not result in Th1 lung immunity after the OVA challenge, although it increased OVA-specific IgG2c antibodies, showing a dichotomy between cellular and humoral immune responses. This difference might be related to the emergence of distinctive T helper cell populations such as T follicular helper cells, related with B cell help in lymphoid organs versus activated/effector Th1 cells that mediate tissue inflammation. However, we also found increased production of IgG2c in IL-12 and IFNγ-KO mice (data not shown) as well as in IL-10/IL12-KO mice, indicating that IgG2c is not solely regulated by the IL-12/IFNγ.

In summary, we found that absorption of CpG to Alum blocks Th2 sensitization signaling *via* MyD88 and IL-10 molecules. In WT mice, this type of sensitization results in a non-polarized immune phenotype revealed by the lack of effector/inflammatory T helper cell appearance in the lung, depressed IgE responses, and enhanced OVA-specific IgG2c antibodies. However, in IL-10-KO animals, this type of sensitization results in IFNγ-dominated lung inflammation and further increase in IgG2c production, while in IL-10/IL-12 double-KO mice, IL-17 production in lung predominates. Therefore, depending on both MyD88 pathway activation and IL-10 plus IL-12 production, CpG adsorbed to Alum induces a spectral T helper sensitization, raging from non-polarized to Th1/Th17-like phenotypes.

A number of TLR ligands are currently under development for vaccine formulations or inflammatory disorders treatment (4, 34). In fact, CpG has been extensively studied and shown to be beneficial in mouse and primate models of asthma as well as in human clinical trials (35, 36). Interestingly, it has been reported that administration of allergen adsorbed to Alum reduced allergen-specific IgE antibodies in atopic patients (37). Also, subcutaneous administration of a ragweed-TLR9 agonist vaccine was clinically effective in allergic rhinitis treatment (38). Moreover, a novel TLR9 agonist showed clinical efficacy in persistent allergic asthma (39).

Collectively, our work highlights the molecular pathways and the adjuvant properties of Alum/CpG-based formulation, which can be potentially exploited in the design of vaccines or immunotherapies for allergy or infectious diseases.

## Author Contributions

LM performed the experiments and wrote the paper; RC performed the experiments, analyzed the data, designed the figures, wrote and reviewed the paper; EG performed all the experiments, and FR performed some experiments; EA and VC performed experiments of Supplementary Figures S1 and S2; and MR conceived and designed the experiments, analyzed the data, wrote and reviewed the paper.

## Conflict of Interest Statement

The authors declare that the research was conducted in the absence of any commercial or financial relationships that could be construed as a potential conflict of interest.

## Supplementary Material

Figure S1 **The 129 WT, 129 SvGR (γ receptor)-KO, or 129 ABR (α/β receptor)-KO mice were sensitized with OVA/Alum or OVA/Alum/CpG on days 0 and 7 and challenged with OVA on days 14 and 21**. Samples obtained on day 22. **(A)** Total cell and **(B)** eosinophil cell counts in BAL; **(C)** IL-5 levels of in BAL; **(D)** total IgE and **(E)** OVA-specific IgE serum levels. Values represent the mean ± SD and are representative of three independent experiments. One-way ANOVA: **p* < 0.05; ***p* < 0.01; ****p* < 0.001; *****p* < 0.0001, difference between OVA/Alum and Ova/Alum/CpG (*n* = 5).

Figure S2 **CpG attenuates airway allergic responses independently of indoleamine 2,3-dioxygenase (IDO) activity**. C57BL/6 WT mice were sensitized with OVA/Alum or with OVA/Alum/CpG on days 0 and 7 and challenged with OVA on days 14 and 21. OVA/Alum/CpG groups were treated or not with 1-methyltryptohan (1-MT) incorporated in pellet, or in Alum or given i.p. **(A)** Total cell and **(B)** eosinophil cell counts in BAL, **(C)** kynurenine concentration in the lung; **(D)** total IgE and **(E)** OVA-specific IgE in serum levels. Values represent the mean ± SD and are representative of three independent experiments. One-way ANOVA: **p* < 0.05; ***p* < 0.01; ****p* < 0.001; different from OVA/Alum (*n* = 5).

## References

[1] Brewer JM. (How) do aluminium adjuvants work? Immunol Lett (2006) 102:10–5. 10.1016/j.imlet.2005.08.002 16188325

[2] Pelka K Latz E. Getting closer to the dirty little secret. Immunity (2011) 34:455–8. 10.1016/j.immuni.2011.04.003 21511178 PMC3128920

[3] Marrack P McKee AS Munks MW. Towards an understanding of the adjuvant action of aluminium. Nat Rev Immunol (2009) 9:287–93. 10.1038/nri2510 19247370 PMC3147301

[4] Steinhagen F Kinjo T Bode C Klinman DM. TLR-based immune adjuvants. Vaccine (2011) 29:3341–55. 10.1016/j.vaccine.2010.08.002 20713100 PMC3000864

[5] Russo M Nahori MA Lefort J Gomes E de Castro Keller A Rodriguez D Suppression of asthma-like responses in different mouse strains by oral tolerance. Am J Respir Cell Mol Biol (2001) 24:518–26. 10.1165/ajrcmb.24.5.4320 11350820

[6] DuPage M Bluestone JA. Harnessing the plasticity of CD4(+) T cells to treat immune-mediated disease. Nat Rev Immunol (2016) 16:149–63. 10.1038/nri.2015.18 26875830

[7] Bortolatto J Borducchi E Rodriguez D Keller AC Faquim-Mauro E Bortoluci KR Toll-like receptor 4 agonists adsorbed to aluminium hydroxide adjuvant attenuate ovalbumin-specific allergic airway disease: role of MyD88 adaptor molecule and interleukin-12/interferon-gamma axis. Clin Exp Allergy (2008) 38:1668–79. 10.1111/j.1365-2222.2008.03036.x 18631348

[8] Bortolatto J Mirotti L Rodriguez D Gomes E Russo M. Adsorption of Toll-like receptor 4 agonist to alum-based tetanus toxoid vaccine dampens pro-T helper 2 activities and enhances antibody responses. J Immunol Res (2015) 2015:280238. 10.1155/2015/280238 26380316 PMC4562177

[9] Muller U Steinhoff U Reis LF Hemmi S Pavlovic J Zinkernagel RM Functional role of type I and type II interferons in antiviral defense. Science (1994) 264:1918–21. 10.1126/science.8009221 8009221

[10] Aida Y Pabst MJ. Removal of endotoxin from protein solutions by phase separation using Triton X-114. J Immunol Methods (1990) 132:191–5. 10.1016/0022-1759(90)90029-U 2170533

[11] Braun D Longman RS Albert ML. A two-step induction of indoleamine 2,3 dioxygenase (IDO) activity during dendritic-cell maturation. Blood (2005) 106:2375–81. 10.1182/blood-2005-03-0979 15947091 PMC1895261

[12] Hemmi H Kaisho T Takeda K Akira S. The roles of toll-like receptor 9, MyD88, and DNA-dependent protein kinase catalytic subunit in the effects of two distinct CpG DNAs on dendritic cell subsets. J Immunol (2003) 170:3059–64. 10.4049/jimmunol.170.6.3059 12626561

[13] Volpi C Fallarino F Pallotta MT Bianchi R Vacca C Belladonna ML High doses of CpG oligodeoxynucleotides stimulate a tolerogenic TLR9-TRIF pathway. Nat Commun (2013) 4:1852. 10.1038/ncomms2874 23673637

[14] Chu RS Targoni OS Krieg AM Lehmann PV Harding CV. CpG oligodeoxynucleotides act as adjuvants that switch on T helper 1 (Th1) immunity. J Exp Med (1997) 186:1623–31. 10.1084/jem.186.10.1623 9362523 PMC2199137

[15] Gavett SH O’Hearn DJ Li X Huang SK Finkelman FD Wills-Karp M. Interleukin 12 inhibits antigen-induced airway hyperresponsiveness, inflammation, and Th2 cytokine expression in mice. J Exp Med (1995) 182:1527–36. 10.1084/jem.182.5.1527 7595222 PMC2192202

[16] Ashino S Wakita D Zhang Y Chamoto K Kitamura H Nishimura T. CpG-ODN inhibits airway inflammation at effector phase through down-regulation of antigen-specific Th2-cell migration into lung. Int Immunol (2008) 20:259–66. 10.1093/intimm/dxm138 18156622

[17] Hafner M Zawatzky R Hirtreiter C Buurman WA Echtenacher B Hehlgans T Antimetastatic effect of CpG DNA mediated by type I IFN. Cancer Res (2001) 61:5523–8. 11454702

[18] Nakajima H Nakao A Watanabe Y Yoshida S Iwamoto I. IFN-alpha inhibits antigen-induced eosinophil and CD4+ T cell recruitment into tissue. J Immunol (1994) 153:1264–70. 7913113

[19] Maeda Y Musoh K Shichijo M Tanaka H Nagai H. Interferon-beta prevents antigen-induced bronchial inflammation and airway hyperreactivity in mice. Pharmacology (1997) 55:32–43. 10.1159/000139510 9309799

[20] Hayashi T Beck L Rossetto C Gong X Takikawa O Takabayashi K Inhibition of experimental asthma by indoleamine 2,3-dioxygenase. J Clin Invest (2004) 114:270–9. 10.1172/JCI21275 15254594 PMC449749

[21] Shinkai Y Rathbun G Lam KP Oltz EM Stewart V Mendelsohn M RAG-2-deficient mice lack mature lymphocytes owing to inability to initiate V(D)J rearrangement. Cell (1992) 68:855–67. 10.1016/0092-8674(92)90029-C 1547487

[22] Moore KW de Waal Malefyt R Coffman RL O’Garra A. Interleukin-10 and the interleukin-10 receptor. Annu Rev Immunol (2001) 19:683–765. 10.1146/annurev.immunol.19.1.683 11244051

[23] van Strien RT Engel R Holst O Bufe A Eder W Waser M Microbial exposure of rural school children, as assessed by levels of *N*-acetyl-muramic acid in mattress dust, and its association with respiratory health. J Allergy Clin Immunol (2004) 113:860–7. 10.1016/j.jaci.2004.01.783 15131567

[24] Kline JN Waldschmidt TJ Businga TR Lemish JE Weinstock JV Thorne PS Modulation of airway inflammation by CpG oligodeoxynucleotides in a murine model of asthma. J Immunol (1998) 160:2555–9. 9510150

[25] Kline JN Krieg AM Waldschmidt TJ Ballas ZK Jain V Businga TR. CpG oligodeoxynucleotides do not require TH1 cytokines to prevent eosinophilic airway inflammation in a murine model of asthma. J Allergy Clin Immunol (1999) 104:1258–64. 10.1016/S0091-6749(99)70022-9 10589010

[26] Longhi MP Trumpfheller C Idoyaga C Caskey M Matos I Kluger C Dendritic cells require a systemic type I interferon response to mature and induce CD4+ Th1 immunity with poly IC as adjuvant. J Exp Med (2009) 206:1589–602. 10.1084/jem.20090247 19564349 PMC2715098

[27] Sun J Walsh M Villarino AV Cervi L Hunter CA Choi Y TLR ligands can activate dendritic cells to provide a MyD88-dependent negative signal for Th2 cell development. J Immunol (2005) 174:742–51. 10.4049/jimmunol.174.2.742 15634894

[28] Blander JM Medzhitov R. Regulation of phagosome maturation by signals from toll-like receptors. Science (2004) 304:1014–8. 10.1126/science.1096158 15143282

[29] Blander JM Medzhitov R. Toll-dependent selection of microbial antigens for presentation by dendritic cells. Nature (2006) 440:808–12. 10.1038/nature04596 16489357

[30] West MA Wallin RP Matthews SP Svensson HG Zaru R Ljunggren HG Enhanced dendritic cell antigen capture via toll-like receptor-induced actin remodeling. Science (2004) 305:1153–7. 10.1126/science.1099153 15326355

[31] Yarovinsky F Kanzler H Hieny S Coffman RL Sher A. Toll-like receptor recognition regulates immunodominance in an antimicrobial CD4+ T cell response. Immunity (2006) 25:655–64. 10.1016/j.immuni.2006.07.015 17000122

[32] Jankovic D Caspar P Zweig M Garcia-Moll M Showalter SD Vogel FR Adsorption to aluminum hydroxide promotes the activity of IL-12 as an adjuvant for antibody as well as type 1 cytokine responses to HIV-1 gp120. J Immunol (1997) 159:2409–17. 9278332

[33] Kinjo T Tomaru K Haines DC Klinman DM. The counter regulatory response induced by CpG oligonucleotides prevents bleomycin induced pneumopathy. Respir Res (2012) 13:47. 10.1186/1465-9921-13-47 22708497 PMC3424146

[34] Basith S Manavalan B Lee G Kim SG Choi S. Toll-like receptor modulators: a patent review (2006-2010). Expert Opin Ther Pat (2011) 21:927–44. 10.1517/13543776.2011.569494 21406035

[35] Vollmer J Krieg AM. Immunotherapeutic applications of CpG oligodeoxynucleotide TLR9 agonists. Adv Drug Deliv Rev (2009) 61:195–204. 10.1016/j.addr.2008.12.008 19211030

[36] Hayashi T Raz E. TLR9-based immunotherapy for allergic disease. Am J Med (2006) 119:.e1–6. 10.1016/j.amjmed.2005.12.028 17000223

[37] Francis JN Durham SR. Adjuvants for allergen immunotherapy: experimental results and clinical perspectives. Curr Opin Allergy Clin Immunol (2004) 4:543–8. 10.1097/00130832-200412000-00012 15640697

[38] Creticos PS Schroeder JT Hamilton RG Balcer-Whaley SL Khattignavong AP Lindblad R Immune tolerance network, immunotherapy with a ragweed-toll-like receptor 9 agonist vaccine for allergic rhinitis. N Engl J Med (2006) 355:1445–55. 10.1056/NEJMoa052916 17021320

[39] Beeh KM Kanniess F Wagner F Schilder C Naudts I Hammann-Haenni A The novel TLR-9 agonist QbG10 shows clinical efficacy in persistent allergic asthma. J Allergy Clin Immunol (2013) 131:866–74. 10.1016/j.jaci.2012.12.1561 23384679

