# Supplementary figures and images for: CpG-ODN Shapes Alum Adjuvant Activity Signaling via MyD88 and IL-10
Source: Front Immunol. 2017 Feb 3;8:47. doi: 10.3389/fimmu.2017.00047 (PMC5289984; doi:10.3389/fimmu.2017.00047)

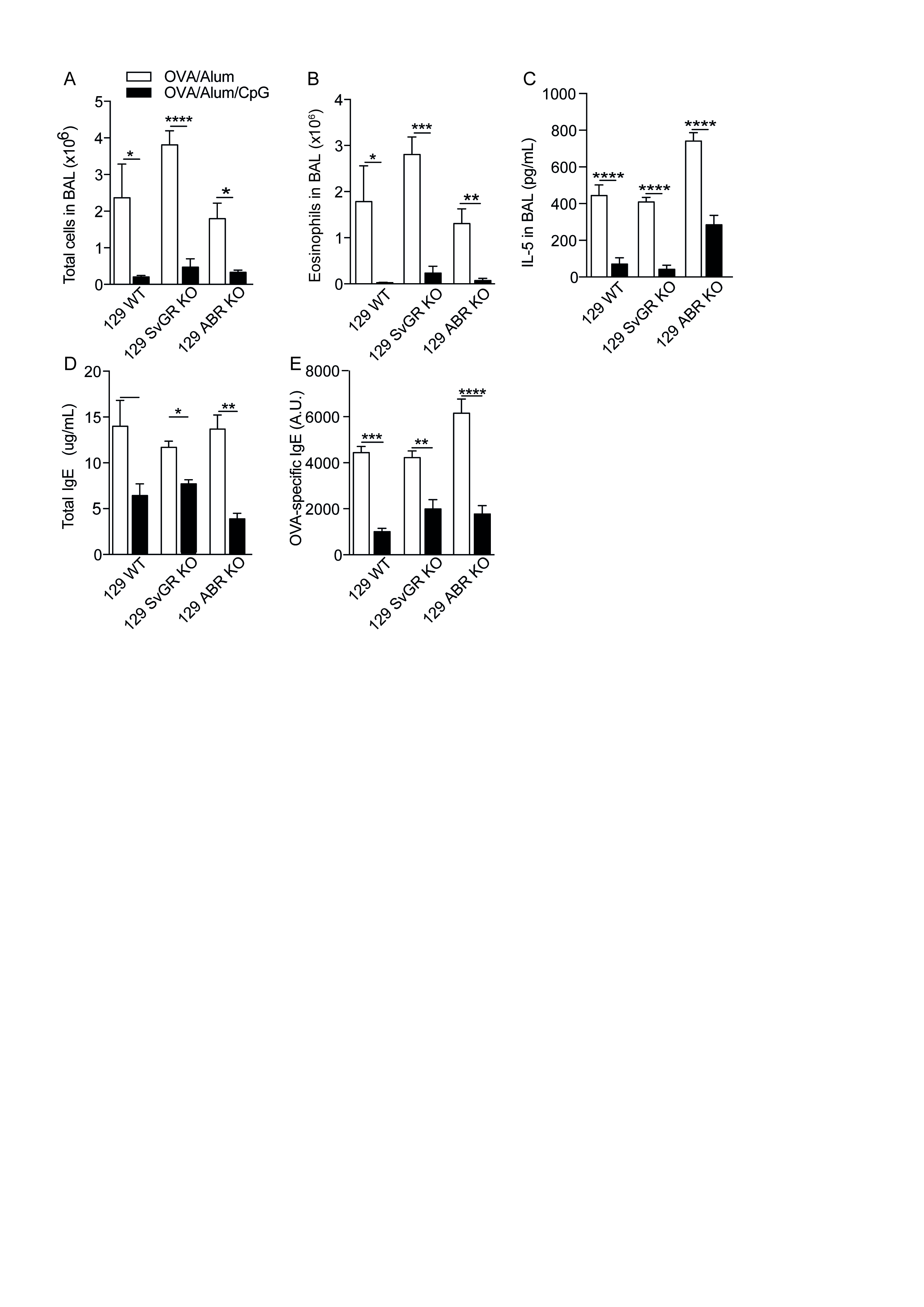

Supplement: Figure S1 — The 129 WT, 129 SvGR (γ receptor)-KO, or 129 ABR (α/β receptor)-KO mice were sensitized with OVA/Alum or OVA/Alum/CpG on days 0 and 7 and challenged with OVA on days 14 and 21. Samples obtained on day 22. (A) Total cell and (B) eosinophil cell counts in BAL; (C) IL-5 levels of in BAL; (D) total IgE and (E) OVA-specific IgE serum levels. Values represent the mean ± SD and are representative of three independent experiments. One-way ANOVA: *p < 0.05; **p < 0.01; ***p < 0.001; ****p < 0.0001, difference between OVA/Alum and Ova/Alum/CpG (n = 5). [file Image_1.TIF]

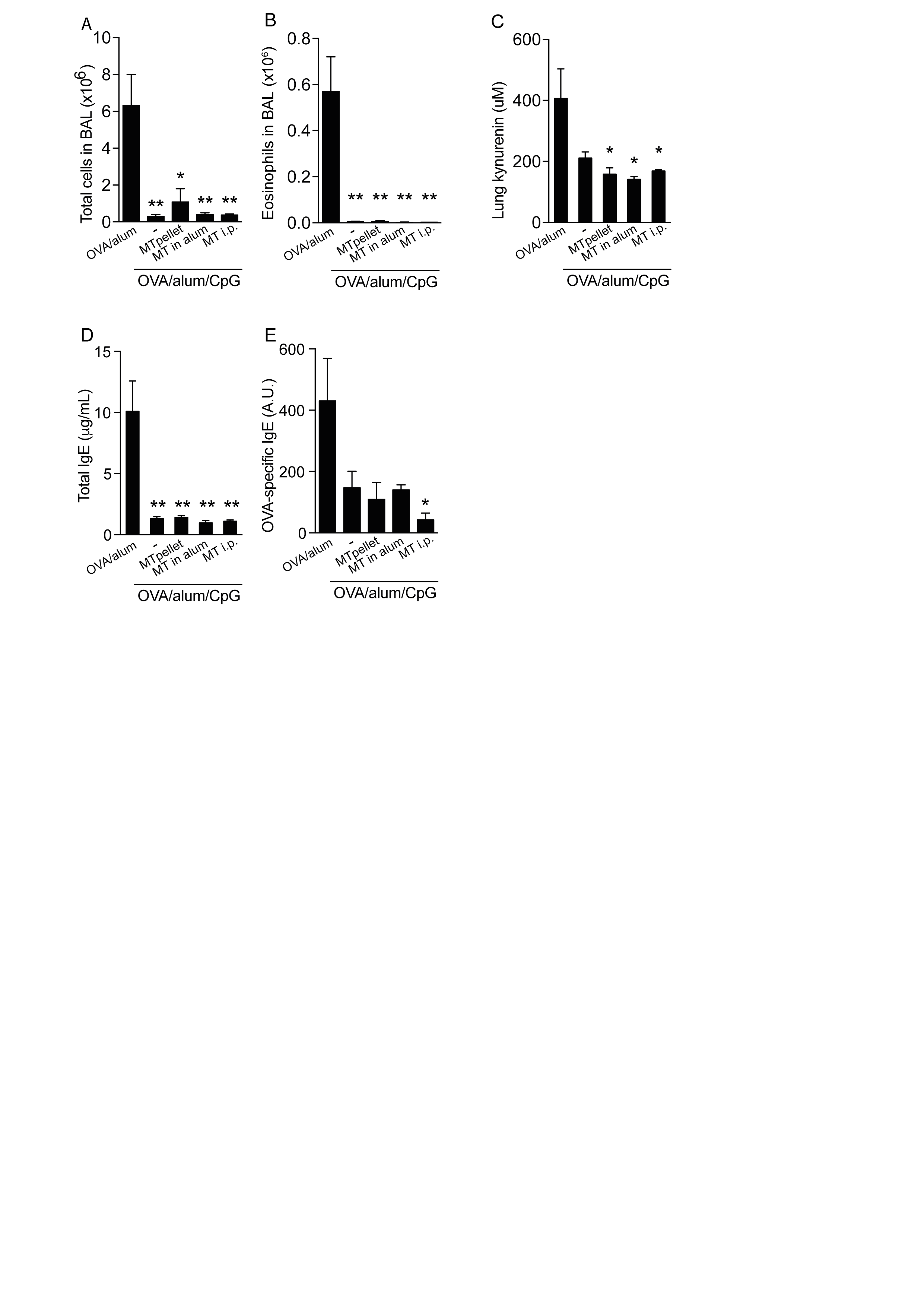

Supplement: Figure S2 — CpG attenuates airway allergic responses independently of indoleamine 2,3-dioxygenase (IDO) activity. C57BL/6 WT mice were sensitized with OVA/Alum or with OVA/Alum/CpG on days 0 and 7 and challenged with OVA on days 14 and 21. OVA/Alum/CpG groups were treated or not with 1-methyltryptohan (1-MT) incorporated in pellet, or in Alum or given i.p. (A) Total cell and (B) eosinophil cell counts in BAL, (C) kynurenine concentration in the lung; (D) total IgE and (E) OVA-specific IgE in serum levels. Values represent the mean ± SD and are representative of three independent experiments. One-way ANOVA: *p < 0.05; **p < 0.01; ***p < 0.001; different from OVA/Alum (n = 5). [file Image_2.TIF]
